# Editorial: Coronary epicardial and microvascular hemodynamics

**DOI:** 10.3389/fcvm.2022.969928

**Published:** 2022-07-22

**Authors:** Alberto Polimeni, Gianluca Campo

**Affiliations:** ^1^Division of Cardiology, Department of Medical and Surgical Sciences, Magna Graecia University, Catanzaro, Italy; ^2^Center for Cardiovascular Research, Magna Graecia University, Catanzaro, Italy; ^3^Cardiology Unit, Azienda Ospedaliero Universitaria di Ferrara, Cona, Italy

**Keywords:** microcirculation, IMR, FFR, RFR, IFR

Recent large trials question the impact of percutaneous coronary interventions in improving patient's prognosis, such that there is a growing need for methods that assess more precisely which patients and lesions deserve intervention.

Physiological assessment of coronary artery disease (CAD) has become one of the cornerstones of decision making for myocardial revascularization. Methods for the invasive or non-invasive assessment of coronary physiology are well established in clinical routine, and their use is steadily expanding. To date, many modalities and indices are available for coronary physiological assessment in the catheterization laboratory.

Of note, most of these modalities used in daily practice focus only on epicardial artery disease, but a substantial number of patients have combined epicardial and microvascular disease. Therefore, it is essential to always take into consideration the presence or absence of microvascular dysfunction and perform appropriate tests (Figure 1) to identify the dominant endotype of coronary microvascular disease.

**Figure 1:**
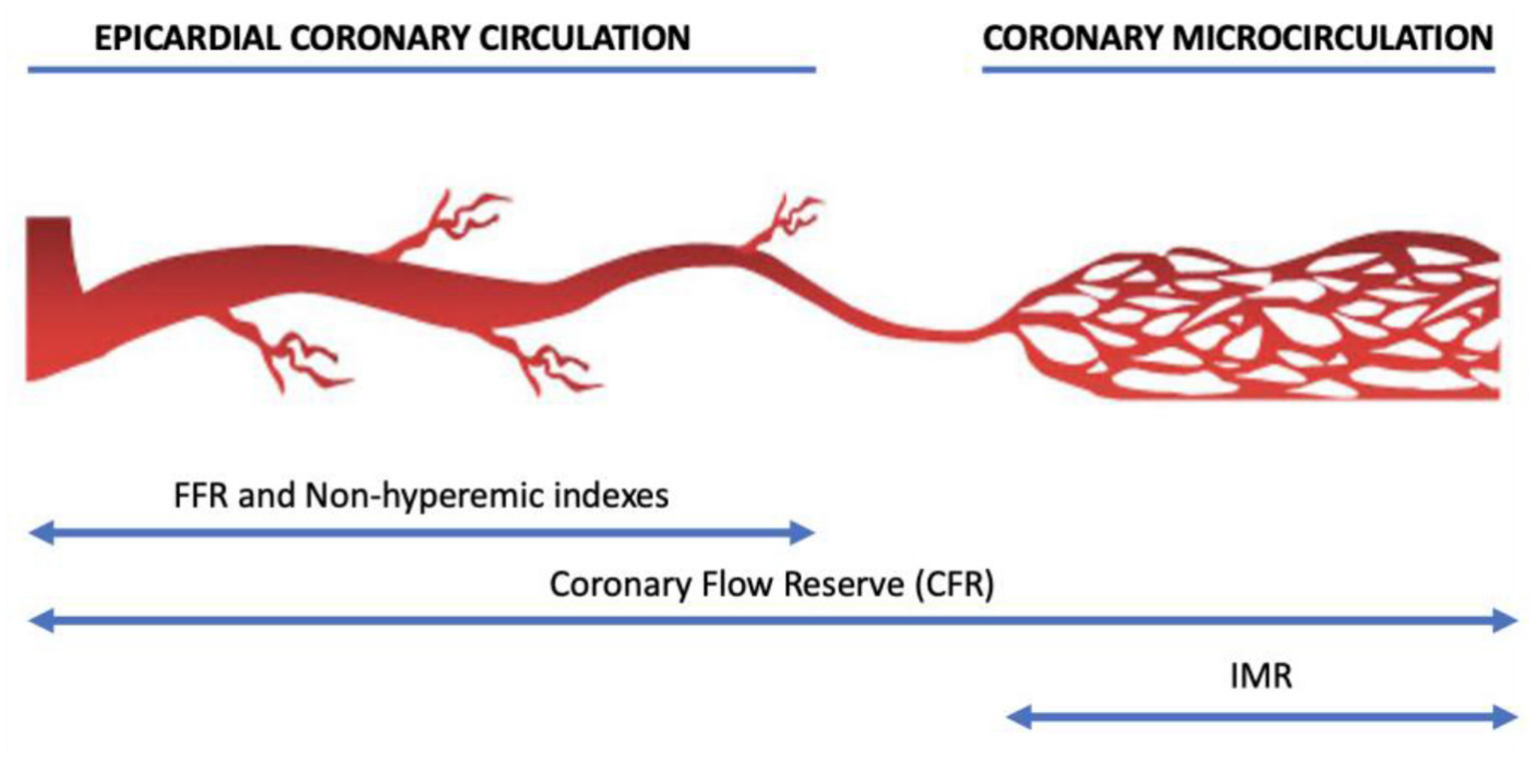
The structure of coronary macrocirculation and microcirculation and the corresponding action fields of physiological techniques.

However, several questions still remain. These pertain, among many other, the relative merits of hyperemia-based vs. resting indexes, the relationship with imaging methods, the impact of post-PCI hemodynamics, scenarios of complex anatomies (e.g., tandem or diffuse stenosis), the role of the microcirculation, the best strategies to treat microvascular dysfunction, the impact of non-invasive or less-invasive technologies and of medical or interventional therapies on coronary hemodynamics.

In the “*Coronary Epicardial and Microvascular Hemodynamics*” Research Topic in Frontiers in Cardiovascular Medicine, several studies underlining the value of coronary physiology were collected.

The purpose of the present anthology is to present the novelties currently available for coronary epicardial and microvascular hemodynamics and discuss the challenges the field is facing to progress further in the next future.

Spione et al. present a complete review on coronary microvascular angina providing an insight into current knowledge of this condition, from current diagnostic methods to the latest treatments.

Li et al. propose a simple and cost-effective index based on corrected TIMI frame count (CTFC) and percent diameter stenosis (DS) to identify flow-limiting coronary stenosis. For this, a new index is put forward as the product of CTFC and DS (PCS). They conclude that PCS is simple and accurate to identify flow-limiting coronary stenosis, especially at vessels with moderate to severe stenosis.

Zuo et al. examine whether quantitative flow ratio (QFR), an angiography-based computation of fractional flow reserve, is associated with intravascular imaging-defined vulnerable plaque features, such as thin cap fibroatheroma (TCFA) in patients with stable angina, and non-ST-segment elevation acute coronary syndrome. They show that lower QFR is related to OCT-defined plaque vulnerability in angiographically mild-to-intermediate lesions and assume the QFR might be a useful tool for ruling out high-risk plaques without using any pressure wire or vasodilator.

Ullrich et al. present a protocol of the randomized trial QUOMODO that will compare quantitative flow ratio or angiography for the assessment of non-culprit lesions in acute coronary syndromes. This study is designed to investigate whether QFR-based decision-making is associated with a decrease in angina and an improved prognosis in patients with multivessel disease.

Schnorbus et al. investigate the impact of antiplatelet therapies on microvascular function in patients undergoing stenting for an acute coronary syndrome. They show that oral loading with prasugrel (and less consistently ticagrelor) is associated with improved microvascular function and stronger platelet inhibition in acute coronary syndrome patient.

Jansen et al. investigate the relation between coronary tortuosity and vasomotor dysfunction in patients without obstructed coronaries showing no association between these two conditions. However, future experimental and clinical studies on the complex interplay between coronary tortuosity, wall shear stress, endothelial dysfunction and coronary flow are warranted.

Yang et al. investigate the influence of coronary disease characteristics on prognostic implications of residual ischemia after coronary stent implantation. They show, in 1476 patients, that coronary disease characteristics including pre-PCI SYNTAX score and pre-PCI FFR affect the prognostic implications of residual ischemia.

Finally, Chen et al. discuss the role of coronary microembolization (CME) as a complication after percutaneous coronary intervention and the impact of miR-200a-3p treatment to protect against CME-induced myocardial injury. This pre-clinical study highlights a novel approach to preventing or treating myocardial damage in clinical settings in the future.

In conclusion, coronary physiology is becoming increasingly important for current interventional cardiologists with robust evidence and an evolving future. Growing novel technologies and modalities in this field are also attracting a great deal of interest.

## Author contributions

AP and GC critically revised the manuscript draft and constructed the figure. Both authors contributed to the article and approved the submitted version.

## Conflict of interest

The authors declare that the research was conducted in the absence of any commercial or financial relationships that could be construed as a potential conflict of interest.

## Publisher's note

All claims expressed in this article are solely those of the authors and do not necessarily represent those of their affiliated organizations, or those of the publisher, the editors and the reviewers. Any product that may be evaluated in this article, or claim that may be made by its manufacturer, is not guaranteed or endorsed by the publisher.

